# Genomic clustering within functionally related gene families in *Ascomycota* fungi

**DOI:** 10.1016/j.csbj.2020.10.020

**Published:** 2020-10-29

**Authors:** Danielle Hagee, Ahmad Abu Hardan, Juan Botero, James T. Arnone

**Affiliations:** Department of Biology, William Paterson University, Wayne, NJ 07470, USA

**Keywords:** Biosynthetic gene clusters, Metabolic gene clusters, Secondary metabolite gene clusters, *Ascomycota* fungi, Genomics

## Abstract

Multiple mechanisms collaborate for proper regulation of gene expression. One layer of this regulation is through the clustering of functionally related genes at discrete loci throughout the genome. This phenomenon occurs extensively throughout *Ascomycota* fungi and is an organizing principle for many gene families whose proteins participate in diverse molecular functions throughout the cell. Members of this phylum include organisms that serve as model systems and those of interest medically, pharmaceutically, and for industrial and biotechnological applications. In this review, we discuss the prevalence of functional clustering through a broad range of organisms within the phylum. Position effects on transcription, genomic locations of clusters, transcriptional regulation of clusters, and selective pressures contributing to the formation and maintenance of clusters are addressed, as are common methods to identify and characterize clusters.

## 1 Introduction

Transcriptional regulation is essential to ensure cellular and organismal survival. Cellular cues, both intracellularly initiated and extracellularly recognized, trigger comprehensive changes to the transcriptome. Such changes establish cellular identity and maintain homeostasis during stress [1], [2], [3], [4], [5], [6]. Transcriptional changes can also be much smaller in scope, allowing for fine-tuning expression of individual genes as needed. At the core of these transcriptional changes is the production of a transient messenger RNA (mRNA) that serves as a template for protein synthesis via the ribosome, commonly referred to as the *Central Dogma of Molecular Biology* [7], [8]. While the *Central Dogma* is a bit of an oversimplification, it remains a foundation for an understanding of gene expression [9], [10]. Extensive study over the decades has yielded a wealth of knowledge about the myriad processes that collaborate to regulate proper gene expression in countless organisms – from the simplest single celled to staggeringly complex [11], [12], [13], [14], [15]. Gene positioning and arrangement throughout the genome can profoundly influence transcription, with the *cis* regulatory ‘logic’ effecting expression across a locus to silence or activate neighboring genes [16], [17]. The influence of genomic arrangement on transcription has yielded significant insights, with implications for all organisms.

**Fig. 1:**
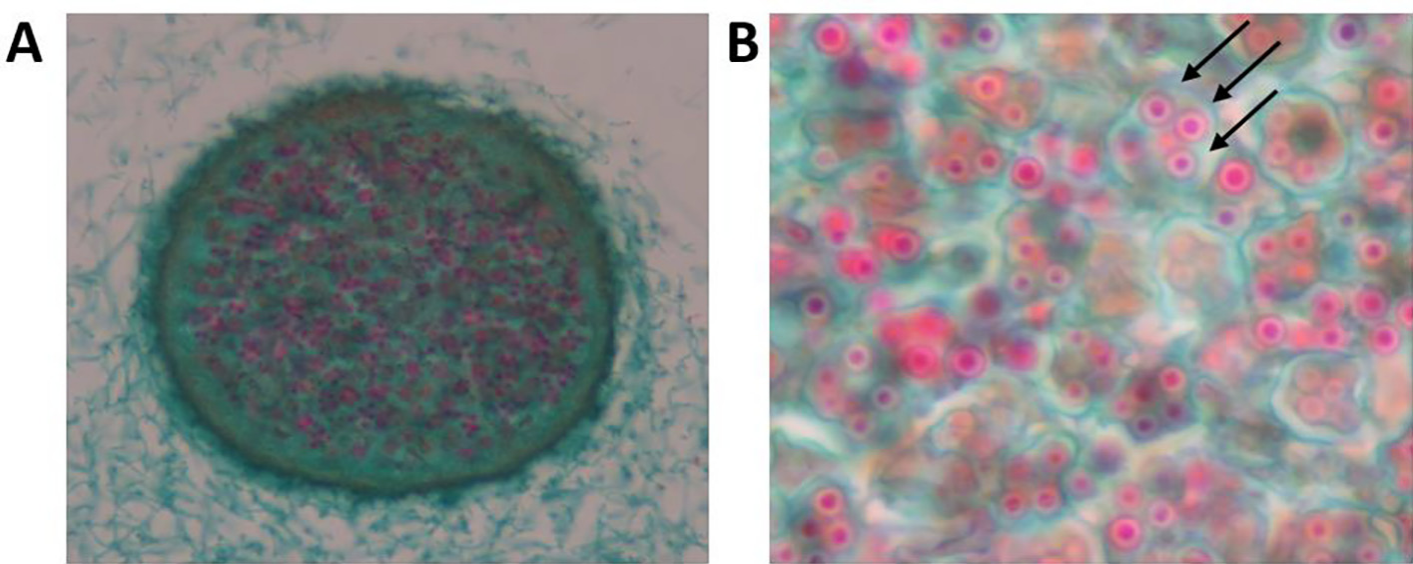
***Penicillium* visualized by light microscopy.** At lower power (A, 400× magnification) the ascocarp (a completely closed fruit body called a cleistothecium) is visible. At higher magnifications (B, 1000× magnification) the ascus is visible with the individual ascospores (three of the spores are highlighted with an arrow). Images courtesy of Kelley Healey.

One of the major clades of living organisms is fungi, with estimates that there may be up to 1.5 million species within this group. There is incredible diversity within the fungal kingdom and throughout the phylum *Ascomycota,* with members exhibiting vast differences in morphology and lifestyles [18], [19]. *Ascomycetes* form a characteristic sac-like structure called an ascus, that forms around their meiotic spores and leads to their common name, sac fungus (Fig. 1) [20]. Representative members are ideal as model organisms for researchers, providing many insights into eukaryotic biology – with multiple Nobel prizes awarded to researchers studying the budding yeast and the fission yeast. It includes filamentous fungi, bread molds, and the causative pathogens for powdery mildew, black rot, and anthracnose [21], [22], [23], [24], [25]. There are also a number of opportunistic human pathogenic *Ascomycete* organisms, including several emerging pathogens [26].

Members of this phylum can have significant metabolic flexibility, which makes them useful for a variety of biotechnological applications, including the production of fatty alcohols, fatty acids, biofuels, reduction and degradation of chemicals and solvents [27]. This phylum includes a broad range of well-studied model organisms, plant pathogens, animal pathogens, and a number of organisms of interest to the pharmaceutical and biotechnology industries (Table 1). Due to the many applications for *Ascomycete* fungi in academia, industry, and medicine, a thorough understanding of genomic organization and the potential implications on gene expression is vital. There have been many advances in the identification and characterization of *Ascomycetes* on a genomic and transcriptional level. In this paper, we review these advances, with a focus on genomic organization and implications upon transcriptional regulation.

**Table 1:** Representative members of the phylum *Ascomycota*Table 1: Representative members of the phylum *Ascomycota.*

	**Species name**	**Note** about species
Model Organisms	*Aspergillus nidulans*	Filamentous fungus
	*Neurospora crassa*	Bread mold
	*Saccharomyces cerevisiae*	Budding yeast
	*Schizzosaccharomyces japonicas*	Fission yeast
	*Schizzosaccharomyces pombe*	Fission yeast
	*Sordaria macrospora*	Model of fruiting body development
Pathogenic and Medically Relevent	*Aspergillus fumigatus*	Aspergillosis
	*Aspergillus terreus*	Potato bilight
	*Aspergillus viridinutans*	Aspergillosis
	*Blastomyces dermatitidis*	Blastomycosis
	*Botrytis cinerea*	Necrotrophic fungus of grapes
	*Candida albicans*	Candidiasis
	*Candida auris*	Candidiasis, high incidence of drug resistance
	*Candida glabrata*	Candidiasis
	*Candida krusei*	Oportunistic pathogen
	*Candida lusitaniae*	Fungemia
	*Candida parapsilosis*	Sepsis
	*Candida rugosa*	Emerging human fungal pathogen
	*Candida tropicalis*	Candidiasis
	*Cladophialphora bantianum*	Cerebral pheohyphomycosis
	*Claviceps purpurea*	Ergot fungus tthat infects rye and cereal and forage plants
	*Coccidioides immitis*	Valley fever (coccidioidomycosis)
	*Coccidioides posadasii*	Valley fever (coccidioidomycosis)
	*Cordyceps militaris*	Entomopathogenic fungus
	*Cryphonectria parasitica*	Chestnut blight (Stajich et al 2009)
	*Dothistroma septosporum*	Red band needle blight
	*Exophiala dermatitidis*	Phaeohyphomycosis
	*Exophiala jeanselmei*	Skin infections
	*Fusarium fujikuroi*	Bakanae disease
	*Fusarium oxysporum*	Animal and plant pathogen
	*Fusarium oxysporum*	Plant and animal pathogen
	*Fusarium solanum*	Onychomycosis, keratomycosis, plant pathogen
	*Gibberella moniliformis*	Maize ear and stalk rot
	*Histoplasma capsulatum*	Pulmonary mycosis
	*Lacazia loboi*	Lobo's disease
	*Leptosphaeria maculans*	Blackleg disease
	*Ochroconis gallopava*	Pathogen to fowls, turkeys, poults, and humans
	*Ophiostoma ulmi*	Dutch elm disease (Stajich et al 2009)
	*Paecilomyces variotii*	Common environmental mold
	*Paracoccidioides brasiliensis*	Paracoccidioidomycosis
	*Paracoccidioides lutzii*	Paracoccidioidomycosis
	*Penicillium expansum*	Psychrophilic blue mold, infects apples
	*Pneumocystis jiroveci*	Pneumocystis pneumonia (PCP)
	*Pseudoallescheria boydii*	Eumycetoma; maduromycosis; pseudallescheriasis
	*Ramichloridium musae*	Banana pathogen
	*Sporothrix schenckii*	Sporotrichosis
	*Talaromyces marneffei*	Talaromycosis (penicilliosis)
Biotechnological and Pharmaceutical Applications	*Aureobasidium pullulans*	Reductase
	*Candida boidinii*	Phenylalanine dehydrogenase
	*Candida cylindracea*	Lipases (Lip) hydrolyze triglycerides into fatty acids and glycerol
	*Candida maltosa*	Resolving D- and L-racemic mixtures of amino acids
	*Candida sorbophila*	ω-oxidizing yeast
	*Debaryomyces hansenii*	Oleaginous yeast; osmotolerant
	*Geotrichum candidum*	Dehydrogenase
	*Kluyveromyces lactis*	Assimilate lactose and convert it into lactic acid
	*Kluyveromyces marxianus*	Aerobic yeast capable of respiro-fermentative metabolism
	*Komagataella pastoris*	Glycolate oxidase; Phytase
	*Ogataea polymorpha*	Protein expression; use methanol consumption
	*Pichia pastoris*	Methanol metabolism
	*Podospora anserina*	Pentaketides
	*Saccharomyces boulardii*	Treatment of intestinal diseases
	*Scheffersomyces stipitis*	Ferment xylose, converting it to ethanol
	*Schwanniomyces occidentalis*	Mylolytic enzymes, including α-amylase and glucoamylase
	*Trichoderma reesei*	Cellulolytic enzymes (cellulases and hemicellulases)
	*Trigonopsis variabilis*	D-amino acid oxidase
	*Yarrowia lipolytica*	Specialty lipids; lipolytic enzymes
	*Zygosaccharomyces rouxii*	L-Glutaminase

*Information from the table compiled and adapted*[22], [25], [26], [27], [28], [126]*.*

## 2 Position effects due to the proximity of heterochromatin

One of the advantages for the usage of members of *Ascomycota* for a wide range of applications is that they are amenable to genetic manipulations [22], [28]. Classical genetic approaches allow for the characterization of molecular functions and frequently utilize reporter genes, or selectable markers, typically utilizing nutritional or drug resistance for selection. The telomere proximal effect (TPE) is a phenomenon well characterized in the budding yeast, *Saccharomyces cerevisiae*, initially identified during the integration of the *URA3*, *TRP1*, and *ADE2* reporter genes. Regardless of genomic orientation, when integrated adjacent to certain telomeres transcriptional repression of the reporter constructs is observed. The TPE is distance dependent; as the region between the telomere and a reporter increased, so does expression of the reporter gene [29]. Transcriptional repression by the TPE is due, in part, to the assembly of transcriptionally inactive heterochromatin that forms at the telomeres and spreads outwards in a continuous fashion mediated by the silent information regulator (SIR) histone deacetylase proteins and the structural maintenance of chromosome (SMC) protein complexes [30], [31].

Further study demonstrated that silencing was not universal at all native yeast telomeres equally, while there are a number of telomeric sites where integration of a reporter gene is heavily silenced there are other sites with little silencing observed [32]. The major indicator of silencing at telomeres appears to be the proximity of the telomere to an autonomously replicating sequence contained within one of the telomeric repetitive elements (the core X element) [32]. A recent study provides the most definitive understanding of TPE via global characterization of transcription utilizing a highly sensitive RNA sequencing approach. There is widely seen transcription at many endogenous yeast telomeres, although at lower absolute levels of transcription compared to non-telomeric regions. Likewise, the SIR proteins play a role in silencing genes at telomeric regions adjacent to known SIR protein binding sites. The majority of telomeric genes are un-effected by the loss of SIR protein function, indicating other mechanisms limiting their expression [33].

The overwhelming majority of eukaryotic organisms maintain heterochromatin at the telomeres. This is true for many *Ascomycetes*, including *Schizosaccharomyces pombe*, *Aspergillus fumigatus*, and *Candida species* [34], [35]. Heterochromatin is a feature of repetitive genomic regions, which include the telomeres, centromeres, and mating loci [36]. While oftentimes heterochromatin conjures images of a static, silent structure, it can be dynamic, exhibiting plasticity under specific growth conditions. *S. pombe* and *C. albicans* alters heterochromatin at the telomere during elevated temperatures in a SIR dependent manner, indicating that this is dynamic – undergoing significant changes depending on the growth conditions [37], [38]. This could result in the clustering of specific genes at specific telomeric regions that require activation under specific stimuli, such as limiting the co-factor necessary for SIR functioning, while maintaining low levels of expression under normal growth conditions [33]. This is particularly relevant for gene members of the toxin response regulon, which is upregulated in response to specific cellular toxins to facilitate cell survival. This family of genes exhibits a non-random distribution throughout the genome, including a number of members localized to the telomeric regions in *S. cerevisiae* [39].

## 3 Functional clustering of genes within complex biosynthetic pathways

Many prokaryotic organisms contain a streamlined genome, with functionally related, co-regulated genes organized in a linear arrangement under the transcriptional regulation of a single promoter region. This organization, called an operon, represents an efficient mechanism to balance production of multiple components within a metabolic process, playing a critical role in gene expression and organismal survival [40], [41], [42], [43]. Operon-like gene clusters are present in at least one eukaryote, although the canonical operon structure is largely absent in eukaryotes on the whole [44].

One characteristic feature of eukaryotic chromosomes is that they exhibit domains, or neighborhoods, of correlated gene expression throughout the genome [45], [46], [47], [48]. In *S. cerevisiae,* there is a broad incidence of locally correlated gene expression, which is distance dependent (the closer any two genes are located the higher their average transcriptional correlation throughout the genome) [49]. This, coupled with the fact that many functionally related genes cluster together non-randomly across the genome, supports the hypothesis that functional clustering represents fundamental layer of transcriptional regulation for many of these genes [39], [45].

Approximately 25% of functionally related gene families are organized into such clusters, and this arrangement increases the transcriptional similarity (as quantified by the Pearson’s correlation coefficient) of these clusters compared to the non-clustered members within the same co-regulated gene family [39]. This phenomenon is not unique to *S. cerevisiae* – clustering of gene families occurs throughout this phylum for gene families participating in a variety of different molecular processes. In this section, we discuss the types and the incidence of these clusters identified in and observed across *Ascomycetes* species.

### 3.1 Secondary metabolite gene clusters

Fungi produce a number of bioactive compounds collectively called secondary metabolites (SMs), molecules that are not required during normal growth. These molecules typically confer a survival advantage exhibiting properties that are antibiotic, anti-proliferative, and catabolic [50]. Many different *Ascomycetes* produce SMs that are mycotoxins, phytotoxins, and compounds that enhance virulence and pathogenesis [51], [52]. The production of SMs by pathogenic fungi can facilitate fungal cooption of a host’s cells, triggering apoptosis and the absorption of host nutrients [52]. Due to the bioactive effects of many SMs, these molecules are of broad interest pharmaceutically and medically for their potentially therapeutic effects [53], [54].

#### 3.1.1 Pathogenic toxins and defense

**Fig. 2:**
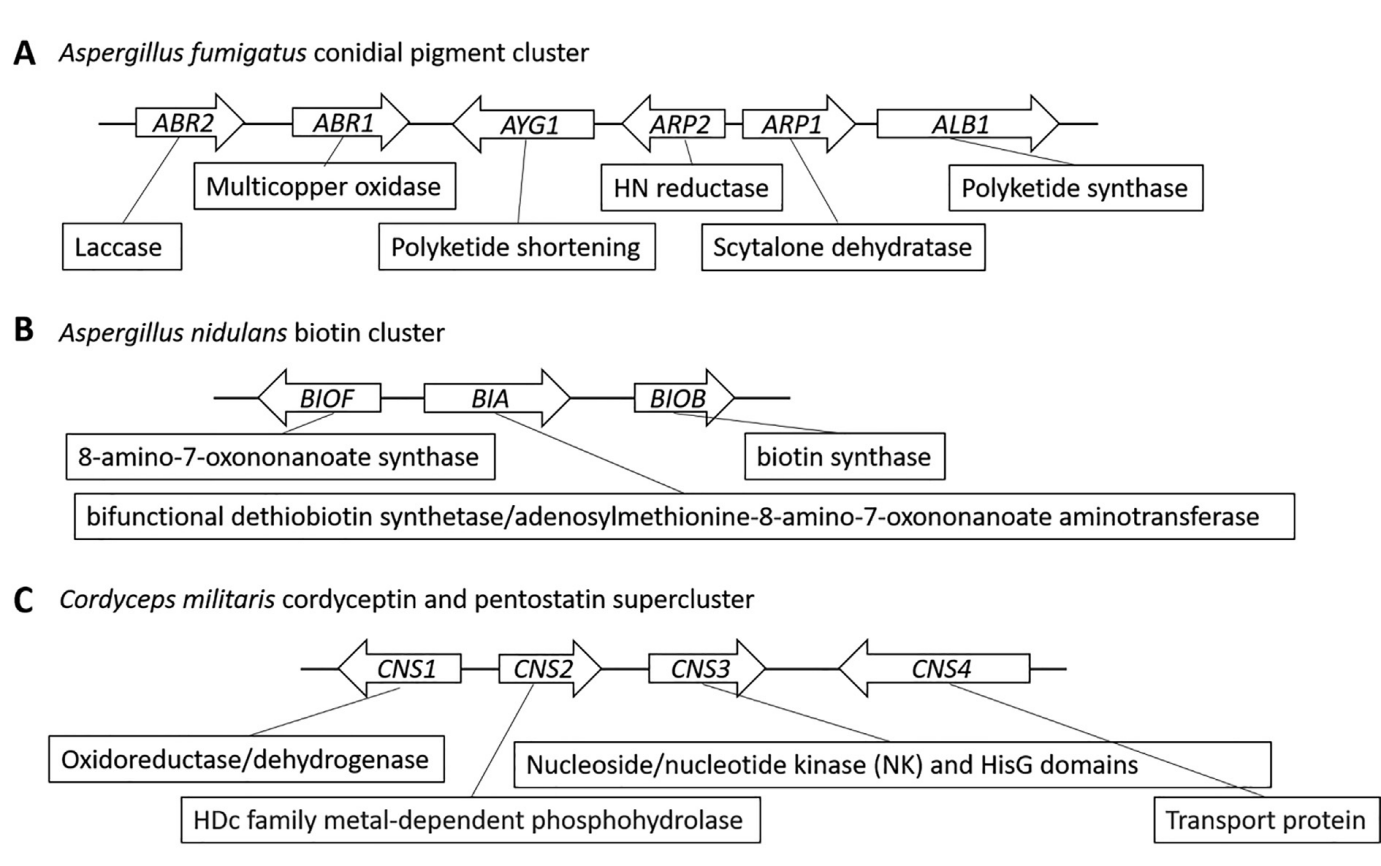
**Genomic arrangement of representative functional clustered biosynthetic genes.** (A) The six-gene cluster in *A. fumigatus* for conidial pigment synthesis, (B) the three-gene cluster in *A. nidulans* for biotin synthesis, and (C) the four-gene supercluster in *C. militaris.* Genomic loci are to approximate scale and each gene is connected to the enzyme that it produces. Data is a compilation from [56], [84], [95]

*Aspergillus* species are non-specific pathogens that infect plants, animals, insects, and immunocompromised people. They cause diseases that cause significant economic impacts in the agricultural industry and produce the potent carcinogen aflatoxin [55]. In *A. nidulans* the pathway for production of sterigmatocystin, a highly toxic metabolite and a precursor to the aflatoxins, is located as a 23-gene cluster that spans a 54 kilobase genomic region. In *A. fumigatus* the six genes which are necessary conidial pigment biosynthesis – known to increase the virulence in this species – form a cluster spanning 19 kb (Fig. 2A) [56]. The biosynthetic pathway for the synthesis of the meroterpenoids austinol and dehydroaustinol in *A. nidulans* are found as a split cluster of four and 10-genes [57].

Gliotoxin is an ETP (epipolythiodioxopiperazine)-type fungal toxin produced by *A. fumigatus* that controls the immune response and induces apoptosis in specific cell types [58]. The biosynthesis of gliotoxin arises from a 16 gene cluster – which includes the synthetic enzymes and the proteins to detoxify and protect *A. fugimatus* from the effects of gliotoxin [59], [60]. This ETP cluster is conserved in many *Aspergillus* lineages. Although there are differences in the clusters between species, this cluster was most likely the result of an ancestral relationship. The current model is that following assembly of this gene cluster they diverged and diversified during the course of evolution of *Ascomycetes* [61].

The clustering of functionally related genes across the genome is widespread within this phylum for genes that are necessary for virulence. The fungal pine pathogen, *Dothistroma septosporum*, produces the potent phytotoxin dothistromin – which is chemically similar to aflatoxin. Biosynthesis of dothistromin requires 19 genes, found in six distinct clusters along the same chromosome [62]. This clustering appears to be the result of a dispersal of an ancestral cluster that exhibited a tighter clustering organizational relationship [62]. *Gibberella moniliformis*, a plant pathogen that causes ‘*bakanae’* disease in rice, contains a cluster of 18 putative genes spanning a 75 kB region for the production of the toxin fumonisin [63].

The rye and grass pathogen, *Claviceps purpurea*, produces ergot alkaloids – potent bioactive mycotoxins – from a four-gene cluster [64]. The antibiotic viriditoxin exhibits anti-proliferative effects in bacteria (via interactions with FtsZ during division) and in cancer cells [65], [66]. This toxin is produced in *Paecilomyces variotii* and *Aspergillus viridinutans* via a nine- and eight- gene cluster respectively [67]. *Penicillium expansum* is a blue mold that causes apple decay and produces the cytotoxic SM patulin, which can be immunosuppressive and carcinogenic [68]. This biosynthetic pathway is encoded by a 15-gene cluster that includes the biosynthetic enzymes and detoxification proteins [69]. *Aspergillus terreus* has a gene cluster for lovastatin biosynthesis, which is used medically to treat high cholesterol and triglyceride levels in patients [70]. Bikaverin is a reddish pigment produced by *Fusarium* fungi with reported antibiotic and antitumoral properties. This molecule depends, in part, on a six-gene cluster as seen in *Fusarium fujikuroi*, where the genes for biosynthesis, regulation, and transport are found in a contiguous stretch [71].

Horizontal gene transfer (HGT) refers to the passage of genes between organisms by means other than parent to offspring transmission. While HGT is quite frequent in prokaryotic organisms, it is significantly less common in eukaryotic organisms. The production of toxins confers a growth and a survival advantage, making SM pathways excellent candidates for HGT in eukaryotes. The 23-gene cluster necessary for sterigmatocystin production in *A. nidulans*, was adopted by the filamentous fungus, *Podospora anserine,* via horizontal gene transfer from *Aspergillus* [72]. Likewise, the biosynthetic clusters for gliotoxin production were acquired by horizontal gene transfer in several species [61]. Interestingly, five of the clustered genes involved in the synthesis of bikaverin in *Fusarium* fungi are identified in the distantly related *Botrytis cinerea*. Although the regulatory genes maintained their functionality, the others did not. This presents an example of horizontal gene transfer of the entire cluster and suggests this mechanism might contribute to the incorporation of novel regulators in addition to metabolic pathways [73].

#### 3.1.2 Detoxification clusters

Arsenic is a naturally occurring element found in a variety of compounds, many of which can be toxic. In budding yeast, *S. cerevisiae*, the three genes that confer resistance to arsenic containing compounds, *ARR1*, *ARR2*, and *ARR3*, are clustered together with a 4.2 kilobase region on chromosome XVI [74]. The expression of these genes results in the production of a basic helix-turn-helix transcription factor, arsenate reductase, and a metalloid-proton antiporter that collaborate to protect the cell from the toxin. *Fusarium oxysporum* contains a two-gene cluster that allows for the detoxification of cyanate (CNO–), a defense compound that is produced by a wide range of organisms that inhibits oxidative phosphorylation through the interference with cytochrome *C* function [75], [76].

In addition to protection from exogenous toxins, there is an inherent risk to produce SMs that are nonspecific toxins. A form of protection for the host cell can involve a gene (or genes) that offers protection from the toxin, termed the ‘resistance hypothesis’. This can include efflux pumps for export and modification or detoxification enzymes [77]. *Leptosphaeria maculans* contains a sirodesmin biosynthesis gene cluster, which is a nonspecific mycotoxin. This gene cluster is predicted to contain 18 genes, including several genes that code for P450 cytochrome proteins that serve to detoxify this compound [78]. The necrotrophic fungus, *Botrytis cinerea*, contains the five-gene cluster for the synthesis of the phytotoxin sequesterterpine and several members of the p450 monooxygenase gene family [79]. It also produces the plant hormone abscisic acid (ABA), coded by a cluster of four genes that include two members of the p450 monooxygenase gene family [80].

### 3.2 Primary metabolite gene clusters

Primary metabolite (PM) biosynthetic genes are those whose products participate in basic metabolic processes that are widespread in many organisms, whereas SMs biosynthetic pathways occur in a limited number of organisms. One of the best characterized functional clustering of a gene family are those of the galactose metabolism genes, which have been extensively characterized in *S. cerevisiae*. Three of the genes are co-localized together on chromosome II: *GAL7*-*GAL10*-*GAL1* which encode the enzymes galactose-1-phosphate uridyl transferase, UDP-glucose-4-epimerase, and galactokinase, respectively [81]. Clustering of the galactose metabolism genes in the *Saccharomycotina* and *Candida* ancestors, with the *S. pombe* and *S. japonicas* acquiring the cluster via HGT from a common ancestor from the *Candida* cluster [82].

Proline catabolism depends on the activity of a permease, an oxidase, and a P5C (pyrroline-5-carboxylate) dehydrogenase to produce glutamate. In *A. nidulans*, proline metabolism is dependent on a four-gene cluster not conserved in *S. cerevisiae*. The biosynthesis of biotin is dependent on a three-gene cluster (Fig. 2B) [83], [84], [85]. Nicotinate metabolism is the result of a six-gene cluster that occurs over a 14.4kB region along chromosome VI in *A. nidulans* [86]. The utilization of alcohol as a carbon source depends on a five-gene cluster found on chromosome VII [87]. Interestingly, there are two additional genes seen in this cluster when compared to the cluster as seen in *A. fumigatus*, representing a species specific expansion of this cluster [87].

*Pichia stipites* contains the genes to catabolize the sugar L-rhamnose, of which four of the five genes are clustered [88]. There at least partial conservation of this grouping in members of the subphyla *Pezizomycotina* and *Saccharomycotina* [88]. Sulfate assimilation depends on the function of an ATP-sulfurase and PAPS (3-phosphoadenoine-5′phosphosulfate) reductase, which are found as a gene pair in *A. terreus* [89].

*C. albicans* catabolize phenolic compounds into acetyl coA via the 3-oxoadipate pathway. The genes involved in this are localized into two distinct clusters and are conserved in the *Candida* and ‘CTG’ lineages (species that translate CUG as serine), although with significant evolutionary divergence [90]. The three genes responsible for GlcNAc metabolism into fructose-6-phosphate are found clustered in *C. albicans*. This arrangement is conserved in *Trichoderma reesei*, along with the transcription factor, *RON1* that regulates expression of the cluster [91].

In the case of PMs, there is an advantage to the clustering of genes; however, it is not a ubiquitous phenomenon. The production of SMs demonstrate an advantage to the maintenance of clusters to maintain integrity of the product and to deal with the potential ramifications of toxicity. This selection does not appear to be the case for PMs which exhibit more variation between species.

### 3.3 Superclusters

The descriptor of superclusters refers to the functional clustering of genes that code for the production of multiple SMs or PMs that have a complex relationship. COR (cordycepin, or 3′-deoxyadenosine) is produced by *Cordyceps militaris* from a four-gene cluster that also includes the enzyme to produce PTN (pentostatin, or 2′-deoxycoformycin), an inhibitor of adenosine deaminases (Fig. 2C). Both of these are bioactive molecules – COR has antibiotic and anti-inflammatory properties and PTN has chemotherapeutic effects [92], [93]. Interestingly, PTN production prevents the deamination of COR to 3′-deoxyinosine by endogenous enzymes *in C. militaris* [94]. COR and PTN biosynthesis is mediated by a single gene cluster and PTN prevents COR deamination, potentially through enzyme inhibition of endogenous adenosine deaminases present in *C. militaris*. Another example of a supercluster is seen in *A. fumigatus*, where the genes for the production of fumagillin and pseurotin are localized to the subtelomeric region of chromosome VIII. There are fifteen genes localized to this cluster, presenting a complex intertwined relationship for these two seemingly unrelated chemicals [95]. This genomic arrangement is conserved, albeit with significant rearrangements in related species [95].

### 3.4 Regulons

While operons are primarily a prokaryotic phenomenon, eukaryotic organisms contain regulons; functionally related gene families that are co-regulated and spread throughout the genome. Canonical examples include the genes involved with the biogenesis of the ribosome – both the ribosomal protein (RP) gene family and the rRNA and ribosome biosynthesis (RRB, or *Ribi*) gene family – are found clustered together throughout the genome in *S. cerevisiae* [96]. This distribution is non-random and highly unlikely to occur by chance [96], [97]. This distribution is not limited to budding yeast, the clustering of both gene families is conserved in both *C. albicans*, and the distantly related *S. pombe*. The identity of the individual paired genes differed among species, however the absolute numbers of clustered genes (e.g. the overall number of pairings that exist) is similar [96], [98]. Systematic analysis revealed that approximately 25% of all functionally related gene families exhibit a non-random genomic distribution as clustered pairings in *S. cerevisiae*. This genomic distribution results in a tighter transcriptional response during cellular changes when compared to the singleton (non-clustered) members within the same family [39].

## 4 Transcriptional co-regulation within a neighborhood may drive functional clustering

In addition to the repressive effects that can occur based on proximity to heterochromatic regions, certain genomic regions are more susceptible to transcriptional disruption during the course of genomic manipulations. The advent of genomic libraries allows for high throughput genetic screening in many organisms, and a wealth of resources exist for *S. cerevisiae*. Genetic manipulation alters expression of genes surrounding the site of manipulation at 7–15% of targeted regions. This led to the misannotation of over nine-thousand genetic interactions in systematic screens [99], [100]. Furthermore, genomic integration sites are responsible for 13-fold variation in protein levels [101]. The disruption of gene expression via the integration of highly expressed reporters may lead to this phenomena through transcriptional interference and repression of the adjacent gene(s) [102], [103].

Aside from transcriptional interference, there is evidence that the non-random genomic distribution of functionally related genes is essential to their proper transcriptional regulation via shared promoter elements. Using a RRB gene pair as a test, functional dissection of the *MPP10-MRX12* pairing identified both genes share a common promoter region. What is notable about this pairing is that the genes are oriented in a convergent manner (→ ←), meaning that the canonical promoter elements, termed PAC and RRPE, exerts influence across a 4.0kB genomic region [104]. Both promoter elements are localized upstream of *MPP10*, there are no identifiable motifs in the promoter region of *MRX12*, and their mutation uncouples transcription of this pair from the rest of the regulon. Transcription of the pair is disrupted by their separation, and proper expression depends on chromatin remodeling and transcription factor binding to the promoter of *MPP10* [105]. This phenomenon is termed ‘adjacent gene co-regulation’ (AGcoR).

**Fig. 3:**
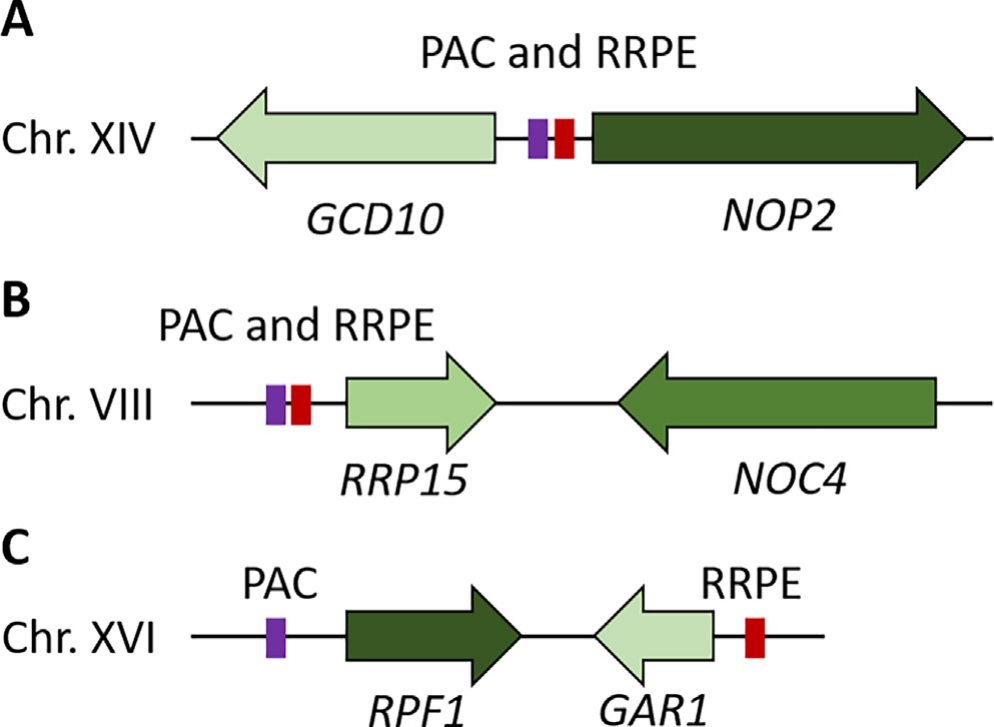
**Representative clustering arrangements observed in *S. cerevisiae* at three RRB paired loci. (**A) The *GCD10-NOP2* locus, (B) the *RRP15-NOC4* locus, and (C) the RPF1-GAR1 locus are shown relative to the PAC and RRPE *cis*- regulatory promoter motifs shown in purple and red, respectively. All loci are to approximate scale, although the relative size of the promoter motifs has been exaggerated to highlight their location and arrangement. Image is . (For interpretation of the references to colour in this figure legend, the reader is referred to the web version of this article.)

There are several conformations for functionally related gene clusters, including a divergent orientation (Fig. 3A). This confirmation results in *cis* regulatory elements between the pair of genes, where it can act as a bidirectional promoter, which is quite common in yeast [106]. Much less common are the tandem (→ →) and convergent orientations (Fig. 3B and C). In these arrangements co-expression and transcriptional regulation must occur across a larger distance – which is not as common in budding yeast [107]. Interestingly, the convergent gene pair *RRP15*-*NOC4* have the PAC and RRPE elements localized upstream of one member of the pair, as in the case with *MPP10*-*MRX12*. The pairing *RPF1*-*GAR1* have the two elements split, with each gene containing either the PAC or the RRPE promoter motif. Though not functionally dissected, it would be quite interesting to observe the transcriptional regulation across a broad genomic distance once it is completed.

adapted from [97]

**Fig. 4:**
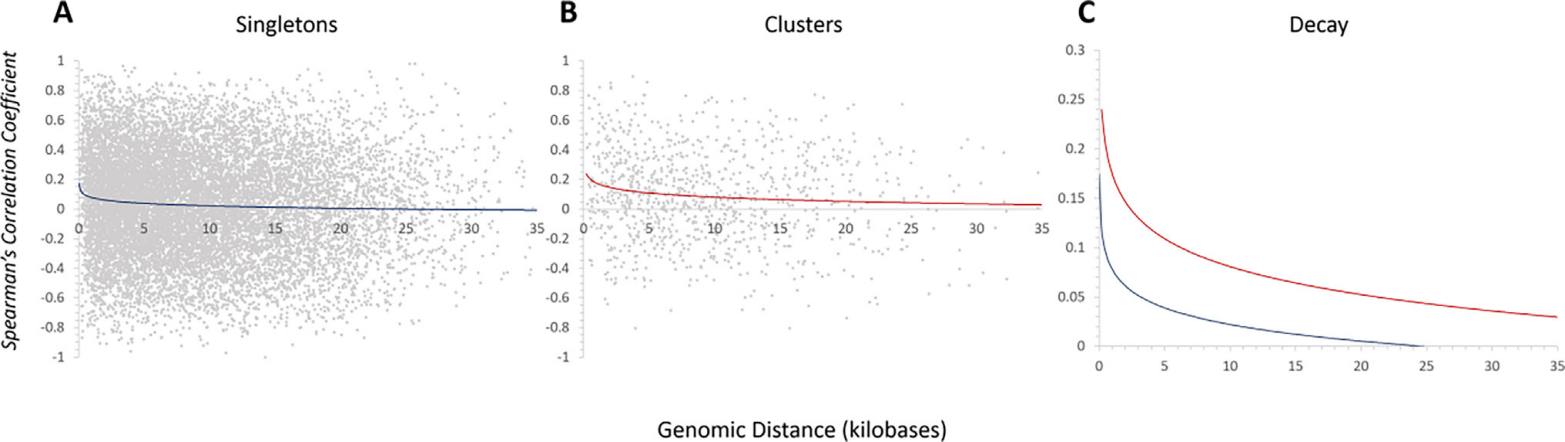
**Functionally clustered genes localize to genomic loci that are more conducive to regulation at a distance.** The pairwise Spearman’s correlation coefficient was determined for the nitrogen metabolism, ribosomal protein, and toxin response genes in *S. cerevisiae* as described [108]. SCC versus genomic distance is plotted for (A) the non-clustered, singleton members of each set and (B) the clustered members of each set and the data was fit to a logarithmic decay. For clarity and ease of comparison, the decay curves are overlaid in (C).

Transcriptional regulation at a distance depends, in part, on alterations to the chromatin underlying a genomic neighborhood extending across a broad region [108]. The constraints that these could impose would result in certain portions of the genome being more permissive to local influencing regulators. This is a particularly attractive model based on the diverse nature of gene families that are clustered and do not necessarily share the same promoter motifs. In support of this model, the ribosomal proteins, the nitrogen metabolism, and the toxin response gene families, cluster into genomic regions that are more susceptible to transcription at a distance during induction of the stress response [108]. Data was extracted and the pairwise Spearman’s correlation coefficient was plotted as a function of genomic distance (between the transcription start sites for each gene) for every member of each family. The members of each family were divided into plots for the singleton members (Fig. 4A) and the clustered members (Fig. 4B). Both plots reveal a positive correlation in expression regardless of genomic confirmation across the stress response. These data fit a logarithmic decay curve, which was overlaid as a separate plot (Fig. 4C). The transcriptional similarity is greater and spreads across a longer genomic distance in the clustered gene members – indicating that regardless of function, clusters are localizing to transcriptionally permissive regions of the genome (at least for these gene families). This would potentially explain how there are similar numbers of clusters within the RP and RRB gene families throughout *Ascomycota*, though the identities of the pairings differ greatly [96].

## 5 Computational approaches to identify and characterize clusters

The identification and characterization of co-regulated functional clusters as described throughout this work is currently an active area of research. This primarily involves two interrelated components – the first is the identification of co-expressed genes and the second is the characterization of the co-localization of two genes to the same genomic locus. In this section, we describe several tools employed to identify correlated expression from gene expression datasets.

### 5.1 Identification of functional clustering relationships using Euclidean distance from gene expression datasets

Identification of the complete membership of co-regulated gene families is a fundamental challenge for researchers, but can yield rewarding applications. One approach that successfully identified the membership of the ribosome biogenesis regulon is through the utilization of the function daisy from the S library [109]. Through the analysis of the budding yeast stress-response datasets, the Euclidean distance for all budding yeast genes (from the composite average gene expression response of the RRB gene family) was determined. This resulted in the expansion of the gene family to 188 members – many of which were clustered throughout the genome and had yet to be annotated [97]. The metric utilized is defined as:

$$d\left(i,j\right)=\sqrt{\sum_{k=0}^{n}{\left(xik-xjk\right)}^{2}}$$

where *x_ik_* and *x_jk_* denote the expression levels of genes *i* and *j* at time point *k* [97]. This approach represents a straightforward method to mine gene expression data when the transcriptional behavior of a gene family is known beforehand.

### 5.2 Pearson’s correlation coefficient analysis of gene expression data

The transcriptional similarity between two genes can be calculated by the Pearson’s correlation coefficient (PCC). This approach is straightforward and represents a standard analysis that can be applied to gene expression data to identify linear relationships among genes, yet powerful enough determine correlated networks and similarities [39]. To calculate the PCC between two genes, *X* and *Y*, across a series of *N* conditions:

$$S\left(X,Y\right)=\frac{1}{N}\sum_{i=1}^{N}\left(\frac{\mathrm{Xi}-Xoffset}{\Phi X}\right)\left(\frac{\mathrm{Yi}-Yoffset}{\Phi Y}\right)$$

where:

$$\Phi G=\sqrt{\sum_{i=1}^{N}\frac{{\left(\mathrm{Gi}-\mathrm{Goffset}\right)}^{2}}{N}}$$

where *G*_offset_ is set to the reference state in each data set.

### 5.3 Spearman’s correlation coefficient analysis of genomic neighborhoods

The Spearman’s correlation coefficient (SCC) is similar to the PCC, although without the magnitude component utilized in the PCC. The use of the SCC is ideal for characterizing gene expression throughout a broad region and uncovering more subtle transcriptional effects within a genomic locus or neighborhood [49]. The SCC is computed utilizing the formula:

$$\rho =\frac{\mathrm{cov}(g1,g2)}{\sigma g1\sigma g2}$$

where *g*1 and *g*2 are the corresponding genes for comparison, cov is their covariance, and σ is the standard deviation of expression. Such analysis can also give an accurate representation of the transcriptional effects that ripple outward as the result of transcriptional activation and that may be indirect as a function of proximity [16], [49]

### 5.4 The multivariate copula model for analysis of gene expression data

An addition to the computational toolkit that allows for modelling the directional dependence of two genes is through the application of a multivariate copula model (MCM) [110]. This represents a powerful computational approach that can help to construct the gene interaction networks and identify co-regulated gene families. This approach, when applied to gene expression datasets, allows for the reconstruction of regulatory relationships even when the data are non-linear [111]. The MCM is an enhancement over the use of the Pearson’s and Spearman’s correlation coefficients that can allow of the characterization of complex networks and relationships – as evidenced by the identification of network of chromosomal maintenance and cell-cycle regulating genes [112].

### 5.5 Determining the statistical significance of genomic arrangement through the with a hypergeometric distribution

Characterization of functional clustering observed in co-expressed gene families has been primarily done via manual curation. While this can be laborious at time, the systematic nomenclature adopted by many organisms allow for rapid searching and querying for identification of clusters. Once the genomic distribution of a gene set has been characterized, the probability of this distribution can be calculated. The statistical significance for the genomic distribution of a functionally related gene family is determined by calculating the binomial probability for the arrangement. The chance probability that there would be *j* adjacent genes within a regulon of size *M* genes is:

$$1-\sum_{k=0}^{j}\left(\frac{M!}{k!\left(M-k\right)!}\right)\left(P^{k}{\left(1-P\right)}^{M-k}\right)$$

where *N* is the total number of genes present within *S. cerevisiae* (total number of genes after deduction of dubious open reading frames).

## 6 Summary and outlook

The physical linkage of genes into functional clusters throughout the genome can buffer the effects of stochastic noise in gene expression. Even in isogenic, clonal populations of cells there can be considerable variation in the levels of expression from similar genetic constructs [113], [114]. One model for selective pressures that favor clustering is that this arrangement would minimize the effects of stochastic noise. This is advantageous when dealing with biological pathways that produce potentially toxic intermediates, as seen with the galactose metabolism and the tyrosine biosynthetic pathways. The clustering of the genes within those pathways can limit variations in the levels of component enzymes – minimizing the risk of toxin buildup [115]. The risk of toxin production is significant, when the *GAL* genes are not clustered in *S. cerevisiae* there is significantly higher levels of the toxic galactose-1-phosphate *and* reduced cell viability [116]. Gene linkage via functional clustering would also minimize copy number variations that would occur during DNA replication, synchronizing the timing together.

A clustering arrangement would also be advantageous when dealing the production of large macromolecular complexes where stoichiometric levels of proteins are necessary. Some of these complexes, such as the ribosome, consume significant energetic reserves [117]. Transcription of the component genes to balance the production and abundance of mRNA as needed, allocates the limited cellular resources to minimize waste [118]. In addition to efficient energetic expenditures, the unbalanced production of the components to produce a ribosome results in orphan ribosomal proteins (RPs). The presence of these extra-ribosomal RPs perturbs cellular proteostasis, activates the expression of the heat shock activated transcription factor, *HSF1*, and reduces cellular fitness [119], [120].

There are thousands of species in the fungal kingdom – and in the phylum *Ascomycota* – yet to be discovered and are still being characterized. There will undoubtedly be many novel SMs identified. Characterization of biosynthetic gene clusters that produce these SMs will provide valuable insight to the target and mechanisms of these SMs, and the defense and protective genes present to ensure secondary metabolites do not harm the host. [121]. This could offer therapeutic targets for emerging pathogens, provide genetic modifications for the protection of food crops, and offer novel pathways for industrial and pharmaceutical biosynthesis. The farther the levels of transcription deviate from the optimal expression level for a gene, the more advantageous the tolerance for transcriptional noise [122]. This is particularly germane for researchers modifying organisms to recreate biosynthetic pathways in organisms that are easier to cultivate, including *S. cerevisiae*. The site of manipulation and modification should be chosen carefully in order to maximize organismal fitness, biosynthetic output, and metabolite yield [123].

The functional clustering of co-regulated genes – and the effects of positional expression – are not limited to *Ascomycetes* or to fungi. It has long been known that the effects of heterochromatin, as in the TPE, are conserved in more complex eukaryotic organisms, including humans [124]. There is conservation of the molecular mechanisms as well [125]. In addition to these repressive effects, there is a global correlation between proximity of two genes and transcriptional similarity [49]. Transcriptional activation of a gene can alter transcription across genomic ‘neighborhoods’, activating expression of the neighboring genes across a large distance [16]. Comprehensive analysis and further elucidation of the mechanisms underlying this phenomena are essential to fully understanding the relationship between the clustering of related genes and co-regulation – and a systems level understanding of transcription within the cell.

## Declaration of Competing Interest

The authors declare that they have no known competing financial interests or personal relationships that could have appeared to influence the work reported in this paper.

## References

[1] Sekhon RS, Lin H, Childs KL, Hansey CN, Buell CR, et al. Genome-wide atlas of transcription during maize development. Plant J 2011;66:553–63. 10.1111/j.1365-313X.2011.04527.x 21299659

[2] Mohammed H., Hernando-Herraez I., Savino A., Scialdone A., Macaulay I., Mulas C., Chandra T., Voet T., Dean W., Nichols J., Marioni J.C., Reik W. (2017). Single-cell landscape of transcriptional heterogeneity and cell fate decisions during mouse early gastrulation. Cell Reports.

[3] Vonk P.J., Ohm R.A. (2018). The role of homeodomain transcription factors in fungal development. Fungal Biol Rev.

[4] Gasch A.P., Spellman P.T., Kao C.M., Carmel-Harel O., Eisen M.B., Storz G., Botstein D., Brown P.O., Silver P.A. (2000). Genomic Expression Programs in the Response of Yeast Cells to Environmental Changes. MBoC.

[5] Storz G., Hengge R. (2010). Bacterial Stress Responses.

[6] Pakos‐Zebrucka K., Koryga I., Mnich K., Ljujic M., Samali A., Gorman A.M. (2016). The integrated stress response. EMBO Rep.

[7] Crick FH. On protein synthesis; 1958, p. 8. 13580867

[8] Crick F. (1970). Central Dogma of Molecular Biology. Nature.

[9] Koonin E.V. (2012). Does the central dogma still stand?. Biol Direct.

[10] Manjrekar J. (2017). Epigenetic inheritance, prions and evolution. J Genet.

[11] Brown J.B., Boley N., Eisman R., May G.E., Stoiber M.H., Duff M.O., Booth B.W., Wen J., Park S., Suzuki A.M., Wan K.H., Yu C., Zhang D., Carlson J.W., Cherbas L., Eads B.D., Miller D., Mockaitis K., Roberts J., Davis C.A., Frise E., Hammonds A.S., Olson S., Shenker S., Sturgill D., Samsonova A.A., Weiszmann R., Robinson G., Hernandez J., Andrews J., Bickel P.J., Carninci P., Cherbas P., Gingeras T.R., Hoskins R.A., Kaufman T.C., Lai E.C., Oliver B., Perrimon N., Graveley B.R., Celniker S.E. (2014). Diversity and dynamics of the Drosophila transcriptome. Nature.

[12] Araya C.L., Kawli T., Kundaje A., Jiang L., Wu B., Vafeados D., Terrell R., Weissdepp P., Gevirtzman L., Mace D., Niu W., Boyle A.P., Xie D., Ma L., Murray J.I., Reinke V., Waterston R.H., Snyder M. (2014). Regulatory analysis of the C. elegans genome with spatiotemporal resolution. Nature.

[13] Stamatoyannopoulos J.A., Snyder M., Hardison R., Ren B., Gingeras T. (2012). An encyclopedia of mouse DNA elements (Mouse ENCODE). Genome Biol.

[14] Consortium EP (2012). An integrated encyclopedia of DNA elements in the human genome. Nature.

[15] Gehring A.M., Walker J.E., Santangelo T.J., Margolin W. (2016). Transcription Regulation in Archaea. J Bacteriol.

[16] Ebisuya M., Yamamoto T., Nakajima M., Nishida E. (2008). Ripples from neighbouring transcription. Nat Cell Biol.

[17] Henikoff S. (1992). Position effect and related phenomena. Curr Opin Genet Dev.

[18] Robbertse B., Reeves J.B., Schoch C.L., Spatafora J.W. (2006). A phylogenomic analysis of the Ascomycota. Fungal Genet Biol.

[19] Schoch CL, Sung G-H, López-Giráldez F, Townsend JP, Miadlikowska J, et al. The Ascomycota tree of life: a phylum-wide phylogeny clarifies the origin and evolution of fundamental reproductive and ecological traits. Systematic biology 2009;58:224–39. 10.1093/sysbio/syp020 20525580

[20] Wallen R.M., Perlin M.H. (2018). An overview of the function and maintenance of sexual reproduction in dikaryotic fungi. Front Microbiol.

[21] Pirrello C, Mizzotti C, Tomazetti TC, Colombo M, Bettinelli P, et al. Emergent Ascomycetes in viticulture: an interdisciplinary overview. Frontiers in Plant Science 2019;10:1394. 10.3389/fpls.2019.01394 PMC6883492 31824521

[22] Duina AA, Miller ME, Keeney JB. Budding yeast for budding geneticists: a primer on the Saccharomyces cerevisiae model system. Genetics 2014;197:33–48. 10.1534/genetics.114.163188 PMC4012490 24807111

[23] Egel R. Fission yeast in general genetics. The Molecular Biology of Schizosaccharomyces pombe: Springer; 2004, p. 1–12.

[24] Pontecorvo G, Roper J, Chemmons L, MacDonald K, Bufton A. The genetics of Aspergillus nidulans. Advances in genetics: Elsevier; 1953, p. 141–238. 10.1016/s0065-2660(08)60408-3 13040135

[25] Davis R.H., Perkins D.D. (2002). Neurospora: a model of model microbes. Nat Rev Genet.

[26] Köhler JR, Casadevall A, Perfect J. The spectrum of fungi that infects humans. Cold Spring Harbor perspectives in medicine 2015;5:a019273. 10.1101/cshperspect.a019273 PMC4292074 25367975

[27] Johnson E.A. (2013). Biotechnology of non-Saccharomyces yeasts—the ascomycetes. Appl Microbiol Biotechnol.

[28] Hoffman C.S., Wood V., Fantes P.A. (2015). An Ancient Yeast for Young Geneticists: A Primer on the Schizosaccharomyces pombe Model System. Genetics.

[29] Gottschling D.E., Aparicio O.M., Billington B.L., Zakian V.A. (1990). Position effect at S. cerevisiae telomeres: reversible repression of Pol II transcription. Cell.

[30] Renauld H., Aparicio O.M., Zierath P.D., Billington B.L., Chhablani S.K., Gottschling D.E. (1993). Silent domains are assembled continuously from the telomere and are defined by promoter distance and strength, and by SIR3 dosage.. Genes Dev.

[31] Moradi-Fard S, Sarthi J, Tittel-Elmer M, Lalonde M, Cusanelli E, et al. Smc5/6 is a telomere-associated complex that regulates Sir4 binding and TPE. PLoS genetics 2016;12:e1006268. 10.1371/journal.pgen.1006268 PMC5001636 27564449

[32] Pryde FE, Louis EJ. Limitations of silencing at native yeast telomeres. EMBO J. 1999;18:2538–50. 10.1093/emboj/18.9.2538 PMC1171335 10228167

[33] Ellahi A., Thurtle D.M., Rine J. (2015). The Chromatin and Transcriptional Landscape of Native Saccharomyces cerevisiae Telomeres and Subtelomeric Domains. Genetics.

[34] De ALP, Juárez-Cepeda J, López-Fuentes E, Briones-Martín-Del-Campo M, Gutiérrez-Escobedo G, et al. Local and regional chromatin silencing in Candida glabrata: consequences for adhesion and the response to stress. FEMS yeast research 2015:15. 10.1093/femsyr/fov056 26122277

[35] Buscaino A. Chromatin-Mediated Regulation of Genome Plasticity in Human Fungal Pathogens. Genes 2019;10:855. 10.3390/genes10110855 PMC6896017 31661931

[36] Bühler M, Gasser SM. Silent chromatin at the middle and ends: lessons from yeasts. EMBO J. 2009;28:2149-61. 10.1038/emboj.2009.185 PMC2722250 19629038

[37] Freire-Benéitez V., Price R.J., Tarrant D., Berman J., Buscaino A. (2016). Candida albicans repetitive elements display epigenetic diversity and plasticity. Sci Rep.

[38] Ayoub N., Goldshmidt I., Cohen A. (1999). Position effect variegation at the mating-type locus of fission yeast: a cis-acting element inhibits covariegated expression of genes in the silent and expressed domains. Genetics.

[39] Eldabagh RS, Mejia NG, Barrett RL, Monzo CR, So MK, et al. Systematic identification, characterization, and conservation of adjacent-gene coregulation in the budding yeast Saccharomyces cerevisiae. Msphere 2018:3. 10.1128/mSphere.00220-18 PMC6001612 29898982

[40] Jacob F, Monod J. Genetic mapping of the elements of the lactose region in Escherichia coli. Biochemical and Biophysical Research Communications 1965;18:693–701.

[41] Kolter R., Yanofsky C. (1982). Attenuation in Amino Acid Biosynthetic Operons. Annu. Rev. Genet..

[42] Osborn A.M., Bruce K.D., Strike P., Ritchie D.A. (1997). Distribution, diversity and evolution of the bacterial mercury resistance (mer) operon. FEMS Microbiol Rev.

[43] Ben Fekih I, Zhang C, Li YP, Zhao Y, Alwathnani HA, et al. Distribution of arsenic resistance genes in prokaryotes. Frontiers in microbiology 2018;9:2473. 10.3389/fmicb.2018.02473 PMC6205960 30405552

[44] Blumenthal T., Davis P., Garrido-Lecca A. (2018). Operon and non-operon gene clusters in the C. elegans genome. WormBook: The Online Review of C elegans Biology [Internet]. WormBook..

[45] Cohen B.A., Mitra R.D., Hughes J.D., Church G.M. (2000). A computational analysis of whole-genome expression data reveals chromosomal domains of gene expression. Nat Genet.

[46] Schmid M., Davison T.S., Henz S.R., Pape U.J., Demar M., Vingron M., Schölkopf B., Weigel D., Lohmann J.U. (2005). A gene expression map of Arabidopsis thaliana development. Nat Genet.

[47] Spellman P.T., Rubin G.M. (2002). Evidence for large domains of similarly expressed genes in the Drosophila genome. J Biol.

[48] Williams E.J., Bowles D.J. (2004). Coexpression of neighboring genes in the genome of Arabidopsis thaliana. Genome Res.

[49] Quintero-Cadena P., Sternberg P.W. (2016). Enhancer Sharing Promotes Neighborhoods of Transcriptional Regulation Across Eukaryotes. G3.

[50] Vining L.C. (1990). Functions of Secondary Metabolites. Annu Rev Microbiol.

[51] Fox E.M., Howlett B.J. (2008). Secondary metabolism: regulation and role in fungal biology. Curr Opin Microbiol.

[52] Möbius N., Hertweck C. (2009). Fungal phytotoxins as mediators of virulence. Curr Opin Plant Biol.

[53] Jensen P.R. (2016). Natural Products and the Gene Cluster Revolution. Trends Microbiol.

[54] Stone M.J., Williams D.H. (1992). On the evolution of functional secondary metabolites (natural products). Mol Microbiol.

[55] Klich M.A. (2007). Aspergillus flavus: the major producer of aflatoxin. Mol Plant Pathol.

[56] Tsai H.-F., Wheeler M.H., Chang Y.C., Kwon-Chung K.J. (1999). A Developmentally Regulated Gene Cluster Involved in Conidial Pigment Biosynthesis in Aspergillus fumigatus. J Bacteriol.

[57] Lo H.-C., Entwistle R., Guo C.-J., Ahuja M., Szewczyk E., Hung J.-H., Chiang Y.-M., Oakley B.R., Wang C.C.C. (2012). Two Separate Gene Clusters Encode the Biosynthetic Pathway for the Meroterpenoids Austinol and Dehydroaustinol in Aspergillus nidulans. J Am Chem Soc.

[58] Scharf D.H., Heinekamp T., Remme N., Hortschansky P., Brakhage A.A., Hertweck C. (2012). Biosynthesis and function of gliotoxin in Aspergillus fumigatus. Appl Microbiol Biotechnol.

[59] Gardiner DM, Howlett BJ. Bioinformatic and expression analysis of the putative gliotoxin biosynthetic gene cluster of Aspergillus fumigatus. FEMS Microbiol Lett. 2005;248:241–8. 10.1016/j.femsle.2005.05.046 15979823

[60] Schrettl M, Carberry S, Kavanagh K, Haas H, Jones GW, et al. Self-protection against gliotoxin—a component of the gliotoxin biosynthetic cluster, GliT, completely protects Aspergillus fumigatus against exogenous gliotoxin. PLoS Pathog 2010;6:e1000952. 10.1371/journal.ppat.1000952 PMC2883607 20548963

[61] Patron NJ, Waller RF, Cozijnsen AJ, Straney DC, Gardiner DM, et al. Origin and distribution of epipolythiodioxopiperazine (ETP) gene clusters in filamentous ascomycetes. BMC Evolutionary Biology 2007;7:174. 10.1186/1471-2148-7-174 PMC2045112 17897469

[62] Bradshaw RE, Slot JC, Moore GG, Chettri P, de Wit PJ, et al. Fragmentation of an aflatoxin‐like gene cluster in a forest pathogen. New Phytologist 2013;198:525–35. 10.1111/nph.12161 23448391

[63] Proctor R.H., Brown D.W., Plattner R.D., Desjardins A.E. (2003). Co-expression of 15 contiguous genes delineates a fumonisin biosynthetic gene cluster in Gibberella moniliformis. Fungal Genet Biol.

[64] Tudzynski P., Hölter K., Correia T., Arntz C., Grammel N., Keller U. (1999). Evidence for an ergot alkaloid gene cluster in Claviceps purpurea. Mol Gen Genet.

[65] Wang J., Galgoci A., Kodali S., Herath K.B., Jayasuriya H., Dorso K., Vicente F., González A., Cully D., Bramhill D., Singh S. (2003). Discovery of a Small Molecule That Inhibits Cell Division by Blocking FtsZ, a Novel Therapeutic Target of Antibiotics. J Biol Chem.

[66] Park J.H., Noh T.H., Wang H., Kim N.D., Jung J.H. (2015). Viriditoxin Induces G2/M Cell Cycle Arrest and Apoptosis in A549 Human Lung Cancer Cells. Nat Prod Sci.

[67] Urquhart A.S., Hu J., Chooi Y.-H., Idnurm A. (2019). The fungal gene cluster for biosynthesis of the antibacterial agent viriditoxin. Fungal Biol Biotechnol.

[68] Moake M.M., Padilla-Zakour O.I., Worobo R.W. (2005). Comprehensive Review of Patulin Control Methods in Foods. Comp Rev Food Sci Food Safety.

[69] Artigot MP, Loiseau N, Laffitte J, Mas-Reguieg L, Tadrist S, et al. Molecular cloning and functional characterization of two CYP619 cytochrome P450s involved in biosynthesis of patulin in Aspergillus clavatus. Microbiology 2009;155:1738. 10.1099/mic.0.024836-0 PMC2889413 19383676

[70] Kennedy J., Auclair K., Kendrew S., Park C., Vederas J. (1999). Modulation of polyketide synthase activity by accessory proteins during lovastatin biosynthesis. Science (New York, NY).

[71] Limón M.C., Rodríguez-Ortiz R., Avalos J. (2010). Bikaverin production and applications. Appl Microbiol Biotechnol.

[72] Slot J.C., Rokas A. (2011). Horizontal Transfer of a Large and Highly Toxic Secondary Metabolic Gene Cluster between Fungi. Curr Biol.

[73] Campbell MA, Rokas A, Slot JC. Horizontal transfer and death of a fungal secondary metabolic gene cluster. Genome biology and evolution 2012;4:289–93. 10.1093/gbe/evs011 PMC3318441 22294497

[74] Bobrowicz P., Wysocki R., Owsianik G., Goffeau A., Ułaszewski S. (1997). Isolation of Three Contiguous Genes,ACR1,ACR2 andACR3, Involved in Resistance to Arsenic Compounds in the YeastSaccharomyces cerevisiae. Yeast.

[75] Elmore MH, McGary KL, Wisecaver JH, Slot JC, Geiser DM, et al. Clustering of two genes putatively involved in cyanate detoxification evolved recently and independently in multiple fungal lineages. Genome Biol Evolution 2015;7:789–800. 10.1093/gbe/evv025 PMC4438557 25663439

[76] Jain A., Kassner R. (1984). Cyanate binding to the ferric heme octapeptide from cytochrome c. A model for anion binding to high spin ferric hemoproteins. J Biol Chem.

[77] Tran P.N., Yen M.-R., Chiang C.-Y., Lin H.-C., Chen P.-Y. (2019). Detecting and prioritizing biosynthetic gene clusters for bioactive compounds in bacteria and fungi. Appl Microbiol Biotechnol.

[78] Gardiner DM, Cozijnsen AJ, Wilson LM, Pedras MSC, Howlett BJ. The sirodesmin biosynthetic gene cluster of the plant pathogenic fungus Leptosphaeria maculans. Molecular microbiology 2004;53:1307–18 10.1111/j.1365-2958.2004.04215.x 15387811

[79] Pinedo C., Wang C.-M., Pradier J.-M., Dalmais B., Choquer M., Le Pêcheur P., Morgant G., Collado I.G., Cane D.E., Viaud M. (2008). Sesquiterpene Synthase from the Botrydial Biosynthetic Gene Cluster of the Phytopathogen Botrytis cinerea. ACS Chem Biol.

[80] Siewers V., Kokkelink L., Smedsgaard J., Tudzynski P. (2006). Identification of an Abscisic Acid Gene Cluster in the Grey Mold Botrytis cinerea. AEM.

[81] Johnston M. A model fungal gene regulatory mechanism: the GAL genes of Saccharomyces cerevisiae. Microbiol Rev 1987;51:458. 10.1128/mr.51.4.458-476.1987 PMC373127 2830478

[82] Slot J.C., Rokas A. (2010). Multiple GAL pathway gene clusters evolved independently and by different mechanisms in fungi. Proc Natl Acad Sci.

[83] Jones S.A., Arst H.N., MacDonald D.W. (1981). Gene roles in the prn cluster of Aspergillus nidulans. Curr Genet.

[84] Magliano P., Flipphi M., Sanglard D., Poirier Y. (2011). Characterization of the Aspergillus nidulans biotin biosynthetic gene cluster and use of the bioDA gene as a new transformation marker. Fungal Genet Biol.

[85] Hull E.P., Green P.M., Arst H.N., Scazzocchlo C. (1989). Cloning and physical characterization of the L-proline catabolism gene cluster of Aspergillus nidulans. Mol Microbiol.

[86] Ámon J., Fernández-Martín R., Bokor E., Cultrone A., Kelly J.M., Flipphi M., Scazzocchio C., Hamari Z. (2017). A eukaryotic nicotinate-inducible gene cluster: convergent evolution in fungi and bacteria. Open Biol.

[87] Flipphi M., Sun J., Robellet X., Karaffa L., Fekete E., Zeng A.-P., Kubicek C.P. (2009). Biodiversity and evolution of primary carbon metabolism in Aspergillus nidulans and other Aspergillus spp.. Fungal Genet Biol.

[88] Koivistoinen O.M., Arvas M., Headman J.R., Andberg M., Penttilä M., Jeffries T.W., Richard P. (2012). Characterisation of the gene cluster for l-rhamnose catabolism in the yeast Scheffersomyces (Pichia) stipitis. Gene.

[89] Schierová M., Linka M., Pažoutová S. (2000). Sulfate assimilation in Aspergillus terreus : analysis of genes encoding ATP-sulfurylase and PAPS-reductase. Curr Genet.

[90] Gérecová G, Neboháčová M, Zeman I, Pryszcz LP, Tomáška Ľ, et al. Metabolic gene clusters encoding the enzymes of two branches of the 3-oxoadipate pathway in the pathogenic yeast Candida albicans. FEMS Yeast Research 2015;15. 10.1093/femsyr/fov006 25743787

[91] Kappel L., Gaderer R., Flipphi M., Seidl‐Seiboth V. (2016). The N ‐acetylglucosamine catabolic gene cluster in Trichoderma reesei is controlled by the Ndt80‐like transcription factor RON1. Mol Microbiol.

[92] Liao Y., Ling J., Zhang G., Liu F., Tao S., Han Z., Chen S., Chen Z., Le H. (2015). Cordycepin induces cell cycle arrest and apoptosis by inducing DNA damage and up-regulation of p53 in Leukemia cells. Cell Cycle.

[93] Tuli H.S., Sharma A.K., Sandhu S.S., Kashyap D. (2013). Cordycepin: A bioactive metabolite with therapeutic potential. Life Sci.

[94] Xia Y., Luo F., Shang Y., Chen P., Lu Y., Wang C. (2017). Fungal Cordycepin Biosynthesis Is Coupled with the Production of the Safeguard Molecule Pentostatin. Cell Chem Biol.

[95] Wiemann P., Guo C.-J., Palmer J.M., Sekonyela R., Wang C.C.C., Keller N.P. (2013). Prototype of an intertwined secondary-metabolite supercluster. Proc Natl Acad Sci.

[96] Arnone J.T., McAlear M.A. (2011). Adjacent Gene Pairing Plays a Role in the Coordinated Expression of Ribosome Biogenesis Genes MPP10 and YJR003C in Saccharomyces cerevisiae. Eukaryot Cell.

[97] Wade C.H., Umbarger M.A., McAlear M.A. (2006). The budding yeast rRNA and ribosome biosynthesis (RRB) regulon contains over 200 genes. Yeast.

[98] Arnone J.T., Robbins-Pianka A., Arace J.R., Kass-Gergi S., McAlear M.A. (2012). The adjacent positioning of co-regulated gene pairs is widely conserved across eukaryotes. BMC Genomics.

[99] Ben-Shitrit T., Yosef N., Shemesh K., Sharan R., Ruppin E., Kupiec M. (2012). Systematic identification of gene annotation errors in the widely used yeast mutation collections. Nat Methods.

[100] Atias N, Kupiec M, Sharan R. Systematic identification and correction of annotation errors in the genetic interaction map of Saccharomyces cerevisiae. Nucleic Acids Res 2016;44:e50–e50. 10.1093/nar/gkv1284 PMC4797274 26602688

[101] Wu X.-L., Li B.-Z., Zhang W.-Z., Song K., Qi H. (2017). Genome-wide landscape of position effects on heterogeneous gene expression in Saccharomyces cerevisiae. Biotechnol Biofuels.

[102] Martens J.A., Laprade L., Winston F. (2004). Intergenic transcription is required to repress the Saccharomyces cerevisiae SER3 gene. Nature.

[103] Martens J.A., Wu P.-Y.-J., Winston F. (2005). Regulation of an intergenic transcript controls adjacent gene transcription in Saccharomyces cerevisiae. Genes Dev.

[104] Bosio M.C., Negri R., Dieci G. (2011). Promoter architectures in the yeast ribosomal expression program. Transcription.

[105] Arnone J.T., Arace J.R., Soorneedi A.R., Citino T.T., Kamitaki T.L. (2014). Dissecting the cis and trans elements that regulate adjacent-gene coregulation in Saccharomyces cerevisiae. Eukaryot Cell.

[106] Xu Z., Wei W., Gagneur J., Perocchi F., Clauder-Münster S. (2009). Bidirectional promoters generate pervasive transcription in yeast. Nature.

[107] Dobi K.C., Winston F. (2007). Analysis of transcriptional activation at a distance in Saccharomyces cerevisiae. Mol Cell Biol.

[108] Cera A., Holganza M.K., Hardan A.A., Gamarra I., Eldabagh R.S. (2019). Functionally Related Genes Cluster into Genomic Regions That Coordinate Transcription at a Distance in Saccharomyces cerevisiae. Msphere.

[109] Venables W.N., Ripley B.D. (2013). Modern applied statistics with S-PLUS.

[110] Kim D., Kim J.-M. (2014). Analysis of directional dependence using asymmetric copula-based regression models. J Stat Comput Simul.

[111] Kim J.-M., Jung Y.-S., Soderberg T. (2009). Directional dependence of genes using survival truncated FGM type modification copulas. Commun Statistics-Simulation Computation.

[112] Kim J.-M., Jung Y.-S., Sungur E.A., Han K.-H., Park C. (2008). A copula method for modeling directional dependence of genes. BMC Bioinf.

[113] Elowitz M.B., Levine A.J., Siggia E.D., Swain P.S. (2002). Stochastic gene expression in a single cell. Science.

[114] Blake W.J., Kærn M., Cantor C.R., Collins J.J. (2003). Noise in eukaryotic gene expression. Nature.

[115] McGary K.L., Slot J.C., Rokas A. (2013). Physical linkage of metabolic genes in fungi is an adaptation against the accumulation of toxic intermediate compounds. Proc Natl Acad Sci.

[116] Xu H., Liu J.-J., Liu Z., Li Y., Jin Y.-S. (2019). Synchronization of stochastic expressions drives the clustering of functionally related genes. Sci Adv.

[117] Warner J.R. (1999). The economics of ribosome biosynthesis in yeast. Trends Biochem Sci.

[118] Li G.-W., Burkhardt D., Gross C., Weissman J.S. (2014). Quantifying absolute protein synthesis rates reveals principles underlying allocation of cellular resources. Cell.

[119] Sorger P.K., Pelham H. (1987). Purification and characterization of a heat-shock element binding protein from yeast. EMBO J.

[120] Tye B.W., Commins N., Ryazanova L.V., Wühr M., Springer M. (2019). Proteotoxicity from aberrant ribosome biogenesis compromises cell fitness. Elife.

[121] Keller N.P. (2015). Translating biosynthetic gene clusters into fungal armor and weaponry. Nat Chem Biol.

[122] Duveau F., Hodgins-Davis A., Metzger B.P., Yang B., Tryban S. (2018). Fitness effects of altering gene expression noise in Saccharomyces cerevisiae. Elife.

[123] Arnone J.T. (2020). Genomic Considerations for the Modification of Saccharomyces cerevisiae for Biofuel and Metabolite Biosynthesis. Microorganisms.

[124] Baur J.A., Zou Y., Shay J.W., Wright W.E. (2001). Telomere position effect in human cells. Science.

[125] Tennen R.I., Bua D.J., Wright W.E., Chua K.F. (2011). SIRT6 is required for maintenance of telomere position effect in human cells. Nat Commun.

[126] Kück U., Pöggeler S., Nowrousian M., Nolting N., Engh I. (2009). Sordaria macrospora, a model system for fungal development. Physiology and Genetics.

